# Enriching Genomic Resources and Marker Development from Transcript Sequences of* Jatropha curcas* for Microgravity Studies

**DOI:** 10.1155/2017/8614160

**Published:** 2017-01-05

**Authors:** Wenlan Tian, Dev Paudel, Wagner Vendrame, Jianping Wang

**Affiliations:** ^1^Environmental Horticulture Department, Institute of Food and Agricultural Sciences (IFAS), University of Florida, Gainesville, FL 32610, USA; ^2^Agronomy Department, Institute of Food and Agricultural Sciences (IFAS), University of Florida, Gainesville, FL 32610, USA; ^3^Tropical Research and Education Center, Institute of Food and Agricultural Sciences (IFAS), University of Florida, 18905 SW 280th Street, Homestead, FL 33031-3314, USA; ^4^Agronomy Department, Genetics Institute, Plant Molecular and Cellular Biology Program, Institute of Food and Agricultural Sciences (IFAS), University of Florida, Gainesville, FL 32610, USA; ^5^Center for Genomics and Biotechnology, Key Laboratory of Haixia Applied Plant Systems Biology, Haixia Institute of Science and Technology, Fujian Agriculture and Forestry University, Fuzhou, Fujian 350002, China

## Abstract

Jatropha (*Jatropha curcas* L.) is an economically important species with a great potential for biodiesel production. To enrich the jatropha genomic databases and resources for microgravity studies, we sequenced and annotated the transcriptome of jatropha and developed SSR and SNP markers from the transcriptome sequences. In total 1,714,433 raw reads with an average length of 441.2 nucleotides were generated. De novo assembling and clustering resulted in 115,611 uniquely assembled sequences (UASs) including 21,418 full-length cDNAs and 23,264 new jatropha transcript sequences. The whole set of UASs were fully annotated, out of which 59,903 (51.81%) were assigned with gene ontology (GO) term, 12,584 (10.88%) had orthologs in Eukaryotic Orthologous Groups (KOG), and 8,822 (7.63%) were mapped to 317 pathways in six different categories in Kyoto Encyclopedia of Genes and Genome (KEGG) database, and it contained 3,588 putative transcription factors. From the UASs, 9,798 SSRs were discovered with AG/CT as the most frequent (45.8%) SSR motif type. Further 38,693 SNPs were detected and 7,584 remained after filtering. This UAS set has enriched the current jatropha genomic databases and provided a large number of genetic markers, which can facilitate jatropha genetic improvement and many other genetic and biological studies.

## 1. Introduction

*Jatropha curcas* L. is a diploid (2*n* = 2*x* = 22) perennial plant in the Euphorbiaceae family that can grow up to 5–7 meters tall and live up to 50 years [1, 2]. The species shows a great potential for biofuel production. It originated from Mexico and Central America and is currently widely distributed in the tropical and subtropical areas, including the Americas, Asia, and Africa. Jatropha seeds contain 30–40% oil, which is suitable for producing biodiesel, especially bio jet fuel [3, 4], and has already become a commercial source for biodiesel production in Senegal, Tanzania, India, and the Philippines [5]. However, jatropha is still an undomesticated plant because breeding and genetic improvement programs are limited [6]. In the United States, it is limited to southern regions because of cold sensitivity, in addition to unavailability of suitable germplasm, and limited genomic resources, which are major concerns in jatropha breeding programs. Selection of best plant materials based on important traits, such as high oil content, high yield, cold tolerance, among others is imperative for commercial success in jatropha breeding.

The microgravity environment has unique characteristics that allow studies of its effects on plant cell structure and physiology [7]. Hereditary variations in tomato submitted to microgravity have been reported, likely due to alterations in the genetic expression through mutations and/or differential gene expression [8]. Therefore, microgravity-induced mutations could be explored as a new approach to plant breeding by combining space biology with agricultural breeding technology [9]. Previous studies in space have demonstrated the effects of microgravity on cell growth, signaling pathways, and gene expression [10–13]. Therefore, the exposure of jatropha cells to microgravity could reveal changes, which could be evaluated for their potential in assisting with the genetic improvement of the species and contribute with its domestication as a feasible crop [9]. Four spaceflights experiments with jatropha cells have been performed and data has been collected for evaluation of differential gene expression using microarray analyses (unpublished data). However, before alterations in genetic expression in jatropha can be evaluated through mutations and/or differential expression, comprehensive genomic information is needed.

The whole genome of jatropha has been sequenced using a combination of Sanger, pyrosequencing, and Illumina sequencing methods [14, 15]. The total length of the final genomic sequences is 297.7 Mb which covers 72–78% of the expected whole genome (380~410 Mb) of jatropha. Expressed sequence tags (ESTs) generated from cDNA libraries provide valuable and reliable information for gene discovery and analysis of gene structure and functions. The first attempt of jatropha EST sequencing had generated 13,249 sequences from developing and germinating seeds, which were assembled into 7,283 unisequences, consisting of 1,606 contigs and 5,677 singletons [16]. Genes related to lipid synthesis, degradation, and some proteins related to toxicity of jatropha seeds were identified in this set of ESTs. Sato et al. [15] sequenced whole genome as well as cDNAs from callus and leaf tissues and generated a total of 21,225 unigenes. Natarajan and Parani [17] reported 14,327 new assembled transcripts with 2,589 full-length cDNA and 27 transcripts directly involved in oil biosynthesis. Subsequently, Eswaran et al. [18] reported 1,240 ESTs generated from root cDNA libraries of jatropha grown at different drought conditions. Hirakawa et al. [14] further assembled all these public available jatropha EST sequences together with their own data, resulting in a total of 30,203 genes, 17,575 transposon related genes, and 2,124 putative pseudogenes. With the advent of low-cost next generation sequencing (NGS) technologies, transcriptome analysis through NGS has become a fast and powerful approach for genome-wide gene discovery and molecular marker development [19–21]. Transcriptomics has been used in jatropha to study gene expression profiles related to cold stress [22]; gene expression in seeds [23]; oil metabolism in maturing seeds [24]; impact of cytokinin on inflorescence buds [25]; metabolic pathways in drought tolerance [26]; and apical meristem [27]. Availability of these resources helps in jatropha gene discovery and crop improvement using molecular tools.

Molecular markers have been systematically accelerating breeding programs through genetic diversity analysis, marker-assisted selection, gene discovery, gene mapping, linkage map construction, quantitative trait locus (QTL) analysis, association analysis, gene transformation, and so forth [28–31]. In jatropha, Random Amplified Polymorphic DNA (RAPD) has been widely and successfully applied to study genetic diversity in India, Africa, China, and other countries [32–37]. Amplified Fragment Length Polymorphism (AFLP) and Inter-Simple Sequence Repeat (ISSR) markers have also been utilized for genetic diversity assessment in India, Malaysia, and other countries [32, 37–44]. Simple Sequence Repeat (SSR) markers are advantageous over the above three types of markers because they are highly polymorphic, abundant, codominant, analytically simple, and readily transferable [45]. However, not many SSR markers have been developed in jatropha. In 2010, SSR markers from cassava were used to investigate the genetic diversity from five jatropha accessions collected from different countries [46]. Sharma and Chauhan [36] showed that 70% (211 out of 320 randomly selected) SSRs from castor bean could be amplified in jatropha but only 7.58% showed polymorphism in 49 jatropha genotypes and 8 jatropha species. Laosatit et al. [27] developed 432 expressed sequence tag-Simple Sequence Repeat (EST-SSR) primer pairs from transcriptome of jatropha and related species, of which 269 were polymorphic. Among the various DNA markers, Single Nucleotide Polymorphism (SNP) markers are the most abundant type of sequence variations in genome [33, 47] and are thus more informative than other markers. In 2011, a total of 1,574 high quality SNPs were identified from 11.9 Gb of sequences, suggesting extremely low frequency of SNPs in jatropha [48]. Later on, 2,482 informative SNPs were discovered from jatropha DNA sequences in a pool of 61 genotypes [47].

Limited genetic diversity in jatropha highlights an immediate need to widen germplasm base [48, 49]. The current molecular tools for jatropha germplasm assessment and genetic studies are not fully developed and molecular markers are not saturated on jatropha genome for high throughput genome wide association analysis. The further enriched genomic resources and molecular markers will facilitate the germplasm evaluation and important gene discovery and elevate the breeding programs for jatropha cultivar improvement. Even though a few NGS based transcriptomes were assembled, all the transcripts in jatropha have not been fully assembled due to high dynamics of gene expression and sequence depth limitation [14, 16, 17, 22–24]. Transcriptomes in young leaves and calli presumably cover the transcripts of majority of the functional genes. This study was initiated in order to generate genomic resources and molecular tools that could assist in microgravity studies and help to accelerate jatropha breeding and genetic improvement. The objectives of this study were (1) to enrich the jatropha genomic databases by transcriptome sequencing and annotating the functions and features of the jatropha transcriptome in leaves and calli and (2) to develop and validate SSR and SNP markers in jatropha. Results from this study will provide useful genomic resource for gene expression analyses in jatropha cell cultures submitted to microgravity and consequently may assist in future genetic, genomic, or biological studies in jatropha.

## 2. Materials and Methods

### 2.1. Plant and RNA Extraction for RNAseq

Jatropha plants were grown at the Tropical Research and Education Center (TREC), in Homestead, FL. Leaf and callus samples collected from six jatropha accessions originating from Mexico, Tanzania, India, Indonesia, Costa Rica, and Fiji were subjected to RNA extraction using Plant RNA Reagent kit (Life Technologies, Carlsbad, CA). The total RNA samples were pooled for cDNA library construction.

### 2.2. Plant and DNA Extraction for Marker Validation

Seven jatropha accessions, each from Mexico, Guatemala, Brazil, Tanzania, Mozambique, India, and Indonesia, were used for DNA extraction. The genomic DNA of these seven plants was isolated from 500 mg young leaves using the method described by Dellaporta et al. [50] with slight modification, specifically, that polyvinylpyrrolidone (PVP) was added into the extraction buffer to remove phenolic compounds and an extra precaution was taken prior to the addition of isopropanol to remove lipids and proteins with chloroform.

### 2.3. cDNA Library Preparation and Sequencing

cDNA library preparation, normalization, and sequencing were performed at the Roy J. Carver Biotechnology Center, University of Illinois, Urbana-Champaign, following the procedures described by Lambert et al. [51]. Firstly, messenger RNA was isolated from 20 *μ*g of total RNA using Oligotex kit (Qiagen, Valencia, CA), followed by the cDNA library construction with titanium adaptors (454 Life Sciences, Branford, CT). Secondly, the cDNA library was normalized using the Trimmer Direct Kit (Evrogen, Russia). Finally, this normalized library was sequenced on the 454 Genome Sequencer FLX+ system according to the manufacturer's instructions (454 Life Sciences, Branford, CT).

### 2.4. Sequence De Novo Assembly and Annotation

Raw sequencing data (deposited in NCBI Short Read Archive with ID: SRR998547 and SRR998927) were assembled using GS de novo Assembler, Newbler v2.8 (454 Life Sciences, Roche, Branford, CT 06405) using “-cdna” and “-vt” options on the input “sff” files that assembles the reads after trimming off the adapter and primer sequences. In order to remove the redundant sequences or isoforms, CD-HIT program [52] was used with settings (-c 0.9, -n 8) to collapse the redundant or isoforms of assembled sequences and to form a uniquely assembled sequence (UAS) set. This UAS set was compared with the upgraded genome sequence of jatropha http://www.kazusa.or.jp/jatropha/ release 4.5 [14] using BLAT at 99% identity. The sequences were also compared with the jatropha CDS database which was assembled from all public available EST sequences (57,437 sequences; ftp://ftp.kazusa.or.jp/pub/jatropha/), [14] using BLAST with a cutoff at *e*-value < 10^−6^ in order to identify novel transcripts.

### 2.5. Assembly Verification by Reverse Transcription (RT) PCR

RNA was extracted from leaves of jatropha accession from Mexico using the Plant RNA Reagent kit (Life Technologies, USA) and reverse-transcribed into cDNA using SuperScript III First-Strand Synthesis System (Invitrogen, USA). The assembly by Newbler was validated by PCR amplification of selected contigs. In total, 15 contigs assembled by Newbler were selected. These 15 contigs covered different length ranges: short (<500 bp), medium (500–1000 bp), and long (>1000 bp) (Table B1) (in Supplementary Material available online at https://doi.org/10.1155/2017/8614160). Primers were designed for flanking the junction of two singletons by Primer3 [53]. For the larger contigs, multiple primer pairs covering different regions were designed. The PCR reactions were set up in a total volume of 20 *μ*L, containing 4 *μ*M of each primer, 1 *μ*L of Taq polymerase, 1x PCR buffer, 40 *μ*M Mg^2+^, 5 *μ*M of dNTP, and approximately 50 ng of template cDNA. The following PCR cycling was used for amplification: initial denaturation at 95°C for 1 min; 46 cycles of 95°C for 45 s, 60°C for 45 s, 72°C for 2 min; and a final extension at 72°C for 7 min. The RT-PCR products were separated on 1% agarose gel and visualized by ethidium bromide staining.

### 2.6. Sequence Annotation

The uniquely assembled sequences (UASs) set was annotated by running blast against GenBank non-redundant (nr) protein and nucleotide (nt) databases using BLASTX (-b 20, -v 20, -p blastx, -e 0.00001, -m 7, –d nr) and BLASTN programs (default parameters with -e 0.00001), respectively. Gene ontology (GO) terms were assigned to UASs by Blast2GO program [54]. The BLASTX output file and the UAS file were submitted to the TargetIdentifier web server to predict full-length cDNAs (http://proteomics.ysu.edu/tools/TargetIdentifier.html) [55]. These UASs were then compared against the protein and nucleotide (untranslated region, coding region, and introns) databases of* Arabidopsis thaliana* (http://www.arabidopsis.org/); castor bean (*Ricinus communis*) (TIGR; http://castorbean.jcvi.org/); and cassava (*Manihot esculenta*) [56] (phytozome) using BLAST (-a 8, -b 0, -v 1, -p blastn, -m 0, -d nt). Furthermore, the sequences were subjected to orthology analyses by aligning to Eukaryotic Orthology Groups (KOG, http://www.ncbi.nlm.nih.gov/COG/) using BLAST with a cutoff at *e*-value < 10^−6^. For pathway analysis, the UAS set was mapped to metabolism pathways using the Kyoto Encyclopedia of Genes and Genomes (KEGG) Automatic Annotation Server (KAAS, http://www.genome.jp/kaas-bin/kaas_main) [57] against the* A. thaliana*,* A. lyrata, Vitis vinifera,* and* Oryza sativa japonica* gene data set using the bidirectional best hit (BBH) assignment method. Plant Transcription Factor (PlnTFDB 3.0, http://plntfdb.bio.uni-potsdam.de/v3.0/) [58] database containing 29,473 sequences of plant genes involved in transcriptional control was used to predict the transcription factors by BLAST with a cutoff at *e*-value < 10^−6^.

### 2.7. SSR and SNP Marker Discovery

SSR detection and primer design were performed using a combined script of MIcroSAtellite (MISA) identification tool (http://pgrc.ipk-gatersleben.de/misa) and Primer3 (http://pgrc.ipk-gatersleben.de/misa/primer3.html) [53]. Compound type SSRs were not counted in this study.

For SNP discovery, the trimmed and filtered reads from Newbler were first aligned to the assembled CDS using BWA-SW with default settings [59]. After that, Samtools was used to call the SNPs [60]. The “SAM” files generated from BWA-SW were further converted to its binary format “BAM” and sorted. In the “mpileup” step, options used were “-u -D -g -f.” The information on SNP calling was stored in “VCF” files.

### 2.8. SSR and SNP Marker Validation

A total of 262 SSR markers were selected for validation, including 88 dinucleotide SSRs and 174 trinucleotide SSRs (Table B2). The selection was based on two criteria: similar number of each motif and the length of SSRs were longer than 18 bp. Primers were synthesized by Invitrogen™, Life Technologies. The amplification and polymorphic rates of these SSRs were tested using seven accessions collected from different countries. Accessions from Mexico, Tanzania, India, and Indonesia were the same as those used for transcriptome sequencing, while we chose different accessions from Guatemala, Brazil, and Mozambique. Validation of markers in these new lines will show the power and correctness of these markers. PCR reactions were performed in 10 *μ*L volumes containing 1 *μ*L of 10x PCR buffer; 0.8 *μ*L of Magnesium Chloride (25 mM); 1.2 *μ*L of dNTP (2 mM); 0.5 *μ*L of Taq enzyme; 2 *μ*L of forward and reverse primer (2 mM); 0.5 *μ*L of DNA template (10 ng/*μ*L); and 4 *μ*L of double distilled water. The PCR program was as follows: initial denaturation at 95°C for 1 min; 5 cycles of 95°C for 45 s, 68°C for 45 s, and 72°C for 40 s; 5 cycles of 95°C for 45 s, 65°C for 45 s, and 72°C for 40 s; 5 cycles of 95°C for 45 s, 60°C for 45 s, and 72°C for 40 s; 5 cycles of 95°C for 45 s, 55°C for 45 s, and 72°C for 40 s; and final extension at 72°C for 7 min. PCR products were separated on 6% nondenatured polyacrylamide gel electrophoresis (PAGE) under 350 volts for 2.5 hours and imaged using silver staining.

Validation of the SNPs was performed by sequencing the amplicons of selected SNP regions using the Sanger method. A total of 21 SNP primer pairs were designed from flanking sequences of the targeted SNPs using Primer3 with two criteria: the amplicon sizes ranged from 400 to 600 bp, and the SNP was close to the middle of the PCR product (Table B3). All the SNP primers were synthesized by Invitrogen, Life Technologies. A PCR protocol for Phusion® High-Fidelity DNA polymerase (New England Biolabs, Inc.) was used following manufacturer's instructions. The amplification and polymorphism of these SNPs were tested using two jatropha genotypes: one from Mexico and the other from Brazil. The PCR program was as follows: initial denaturation at 94°C for 3 min; 5 cycles of 94°C for 30 s, 68°C for 20 s, and 72°C for 40 s; 5 cycles of 94°C for 30 s, 65°C for 20 s, and 72°C for 40 s; 5 cycles of 94°C for 30 s, 60°C for 20 s, and 72°C for 40 s; 5 cycles of 94°C for 30 s, 55°C for 20 s, and 72°C for 40 s; and final extension at 72°C for 7 min. The resulting PCR products were purified using Qiagen minElute PCR purification kit (Qiagen, Valencia, CA). The purified PCR products were then sequenced using Sanger method at the Interdisciplinary Center for Biotechnology Research, University of Florida.

## 3. Results

### 3.1. 454 Sequencing and Assembly

**Figure 1:**
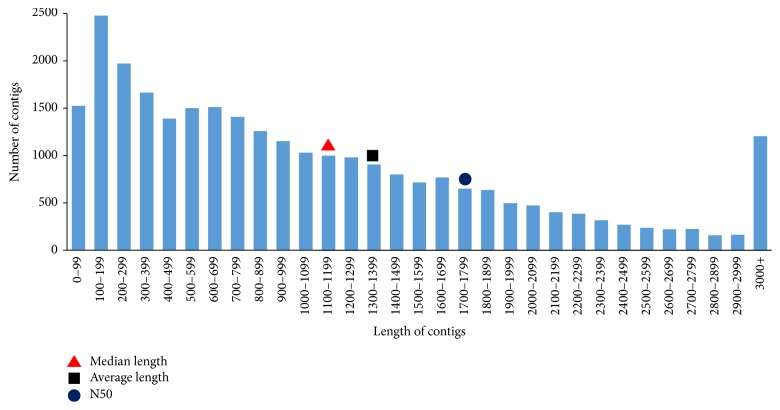
Length distribution of the assembled contigs from Newbler.

**Figure 2:**
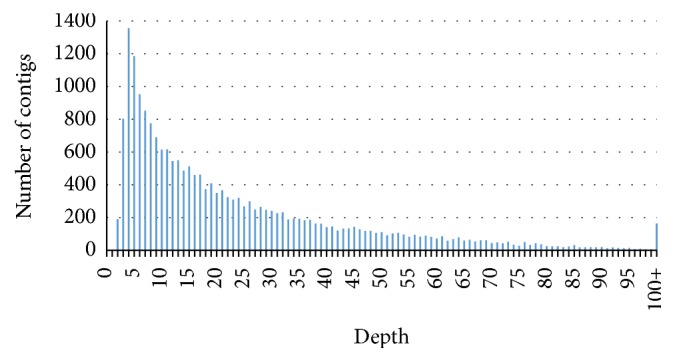
Contig depth distribution.

The cDNA library sequencing runs on 454 GS-FLX platform generated 1,714,433 reads, including 2 lanes of titration reads (48,586 and 44,009) and 2 lanes of sequencing reads (835,663 and 786,175), with an average sequence length of 441.2 nucleotides. After trimming the primers and adapters and removing the low quality reads, 1,714,419 high quality reads were subjected to Newbler v 2.8 assembly. In total, 1,512,952 (88.3%) reads were assembled into 27,897 contigs using Newbler v2.8 (Table 1, Figure 1). In order to get uniquely assembled sequences, the contigs along with singletons and reads that were not assembled were clustered to avoid redundancy and isoforms using CD-HIT [52]. Sequences less than 100 bp were discarded and a total number of 115,611 UAS set was formed. This UAS set included 20,624 contigs after removing 1,526 contigs that were shorter than 100 bp. The length of contigs in the final UAS set ranged from 100 to 11,929 bp, with an average of 1,380.9 nucleotides, a N50 of 1,746 nucleotides (Table 1, Figure 1), and an average depth of 23.6 folds (Figure 2). We compared our UAS set against the upgraded genome sequence of jatropha release 4.5 [14] that consists of 39,277 contigs with a total of 297 Mbp and a N50 of 15,950 bp. This genome sequence covered 71% of the estimated jatropha genome of 416 Mbp [61]. Out of 115,611 sequences in the UAS set, 90,889 sequences (78.61%) showed a significant match on the current jatropha genome.

The final assembly of our UAS set contained a substantial number of large contigs, with the largest contig of 11,929 nucleotides. We picked the top 10 longest contigs to check their assembly and functions individually (Table B4). All of the longest contigs are full-length cDNAs with CDS. The longest protein-coding gene identified was hypothetical protein POPTR_0011s09640g* [Populus trichocarpa]* with 3,755 amino acids. Nine of the ten long contigs specifically aligned to unique proteins with relatively high sequence identity indicating successful assembly of these long contigs and one contig hit multiple protein sequences. RT-PCR and Sanger sequencing of the PCR products were used to verify the fidelity of assembly. For 15 selected contigs assembled by Newbler v2.8,  16 primer pairs were designed. All of the primer pairs from the target contigs amplified the cDNA fragments with expected size, except one with a smaller amplicon size (Table B1). Out of the 115,611 UAS set, 21,418 were identified as full-length cDNAs with complete Open Reading Frame (ORF).

**Figure 3:**
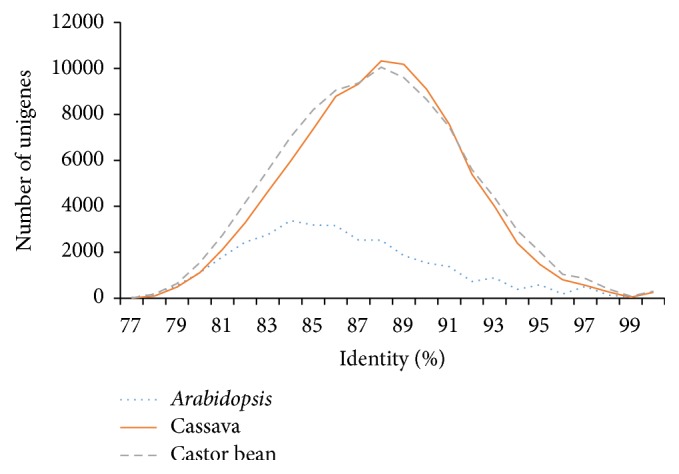
Gene index distribution for* Arabidopsis*, cassava, and castor bean.

After aligning the whole UAS set to the jatropha CDS database which was assembled from all public available EST sequences (57,437 sequences; ftp://ftp.kazusa.or.jp/pub/jatropha/) [14], a total of 23,264 UASs including 9,301 contigs were identified as novel sequences and they were deposited into NCBI DDBJ/EMBL/GenBank (TSA accession ID: GAOW00000000, first version) to enrich the current jatropha EST database. Further, the UAS set was compared against the NCBI protein and nucleotide databases separately, resulting in a total of 77,807 (67.3%) and 70,431 (60.9%) of the UAS, respectively, having hits indicating significant orthology and homology of this UAS set with previously discovered sequences from several other species. When comparing the UAS set to protein and nucleotide databases of* Arabidopsis*, castor bean, and cassava, respectively (Table B5), it was found that jatropha UASs had a much higher identity to cassava and castor bean as compared to* Arabidopsis* (Figure 3).

### 3.2. Functional Annotation and Classification of the UAS Set

**Figure 4:**
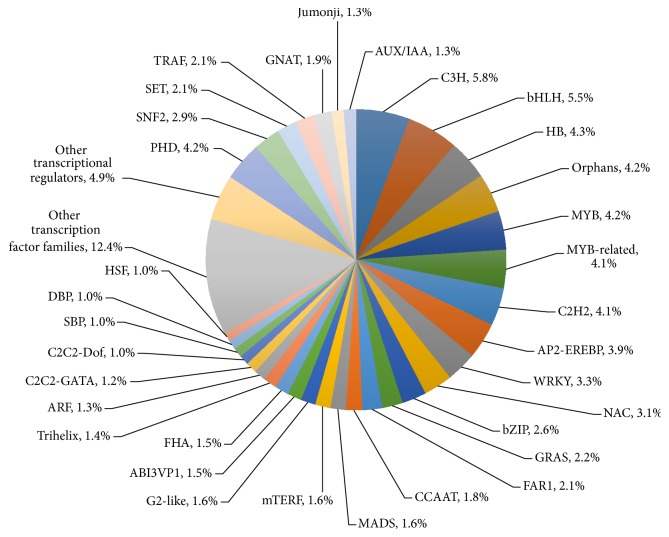
Classification distribution of the transcripts in different transcription factor families.

To understand the functions of the UASs, the whole UAS set was compared to a known transcription factor database and 14,868 (12.9%) UASs were identified as transcription factors, which were distributed in 60 families and 22 other transcription factor regulators. Among the transcription factor families, the C3H family (208, 5.8%) was the most abundant one, followed by bHLH (198, 5.5%), HB (154, 4.3%), orphans (150, 4.2%), MYB (149, 4.2%), MYB-related (148, 4.1%), and PHD (224, 4.2%) transcriptional regulator (Figure 4).

**Figure 5:**
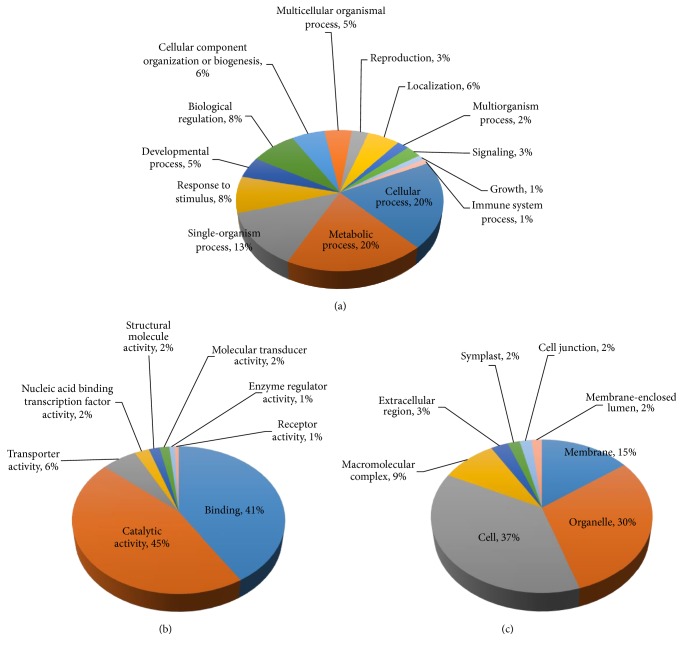
Classification distribution of the annotated GO terms of the UASs.

The UAS set was further classified into functional categories by assigning GO terms using the Blast2GO program. A total of 59,903 annotation counts were obtained and the proportions of each UAS attributing to different functional categories were determined (Figure 5). In the biological processes, cellular (21%) and metabolic processes (20%) constituted the major categories suggesting active cellular and metabolic functions in the jatropha callus and leaves (Figure 5(a)). Among the molecular functions, catalytic (43%) and binding (43%) activities were the two major categories, which are in agreement with the active metabolic functions in the examined tissues (Figure 5(b)). Most of the genes were functioning in cellular (36%) and organelle (30%) components. Some genes were playing functions as membrane components (15%), macromolecular complex components (8%) and other compartments of the cell (Figure 5(c)).

The UAS set was further classified by aligning to the KOG protein database, a highly conserved functional core gene set based on orthologous relationships between genes [62, 63]. In total, 12,584 (10.9%) UASs showed significant similarity to the KOG database belonging to 25 categories. Among the 12,584 UAS sequences, 7,635 (60.7%) were assigned to single or multiple KOG categories (Fig A1) and 1,573 (20.6%) were categorized as general function prediction only. A total of 2,706 (35.4%) genes were involved in cellular process and signaling including posttranslational modification, protein turnover, and chaperones signal transduction mechanisms, and 2,027 (26.5%) of the UASs were involved in metabolism pathways such as transport and metabolism of carbohydrate, lipid, and amino acids. A small portion of the UASs (290, 3.8%) was classified into a group of secondary metabolites biosynthesis, transport, and catabolism, suggesting that those important processes occurred in the leaves and callus of jatropha.

To understand the pathways and gene interactions where the UASs are involved, all the UASs were analyzed in the KEGG pathway database, a collection of manually drawn pathway maps representing knowledge on the molecular interaction and reaction networks. Out of the 115,611 UASs, 8,822 (7.6%) had significant matches in the database and were assigned to six main categories (Fig A2) including 317 different KEGG pathways (Table B6). Most of the mapped genes (3,135; 35.5%) were involved in metabolism, such as carbohydrate metabolism, amino acid metabolism, energy metabolism, nucleotide metabolism, lipid metabolism, and biosynthesis of other secondary metabolites suggesting various metabolic activities of the collected tissues. Some UASs (1,621; 18.4%) were divided into the genetic information processing including transcription, translation, folding, sorting and degradation, replication, and repair. A significant portion of (1,038, 11.8%) UASs were classified into organismal systems containing plant-pathogen interaction, plant circadian rhythm, and natural killer cell mediated cytotoxicity. Moreover, 734 (8.3%) and 657 (7.4%) UASs were involved in cellular process and environmental information processing, respectively.

To explore the applications of this UAS set, we searched for genes involved in biosynthesis of oil, such as fatty acid and triacylglycerol (TAGs) contained in jatropha seed. In the UAS set, biosynthesis pathways of several types of oils including four major fatty acids, oleic, linoleic, palmitic, and stearic fatty acids, were identified. Through KEGG mapping, 23 UASs [13 KEGG orthologs (KOs)] involved in fatty acid biosynthesis, 12 UASs (7 KOs) in fatty acid elongation, and 51 UASs (27 KOs) involved in fatty acid metabolism were identified. In addition, 6 UASs (4 KOs) were found to be involved in linoleic acid metabolism. With respect to the pathways of fatty acid biosynthesis, several UASs were found to encode enzymes such as acetyl-CoA carboxylase, acyl-[acyl-carrier-protein] desaturase, and fatty acyl-ACP thioesterase A/B (Fig A3 and Table 2). Manipulation of these functional genes using biotechnology will help to enhance oil production in jatropha.

Since jatropha is a cold sensitive tropical plant, cold stress responsive genes are of great importance to molecular biologists and breeders. We picked 30 cold responsive genes to check whether they exist in jatropha. These 30 candidate genes included transcription factors like C-repeat- (CRT-) binding factors (CBF1-3), inducer of CBF expression 1 (ICE1, MYC-like basic helix-loop-helix transcription factor), and cold regulated genes (COR) like Rd29a, KIN1, cor15a, and others (Table B7). After BLASTX search, six of the 30 cold stress responsive genes including High Expression of Osmotically Responsive Genes (HOS1), a RING-type ubiquitin E3 ligase, ICE1, and CBF1 had putative orthologs in the jatropha UAS set (Table 3). Three of these genes, namely, HOS1, ICE1, and CBF1, were also found to be differentially expressed during cold stress in jatropha [22].

### 3.3. Characterization of SSRs and Validation

A total of 9,798 SSRs were discovered from the jatropha UAS set, including 6,668 (68.1%) dinucleotide SSRs, 3,047 (31.1%) trinucleotide SSRs, 46 (0.5%) tetranucleotide SSRs, 9 (0.1%) pentanucleotide SSRs, and 29 (0.3%) hexanucleotide SSRs (Table B8). In general, the SSR presence decreased with an increase in the nucleotide number in repeat units. Among the 64 SSR motifs, the most frequent motif type was AG/CT (45.8%), followed by AT/AT (17.0%), AAG/CTT (8.3%), and AC/GT (5.2%). Primer pairs were designed from the flanking sequences of all SSRs except 27 SSRs discovered.

A total of 262 SSR markers were selected for validation based on different motifs, including 88 dinucleotide SSRs and 174 trinucleotide SSRs (Table B2). Validation of these SSRs was based on the size difference among the seven different jatropha genotypes. In total, 202 (77.1%) of the selected 262 SSRs were successfully amplified. For dinucleotide SSRs, a polymorphic rate of 18.3% was observed and for trinucleotide SSRs, 15.5% were polymorphic (Table 4). Interestingly, all the polymorphic SSRs showed unique band pattern in Mexico accession.

### 3.4. SNP Calling and Validation

A total of 38,693 SNPs were detected from the jatropha UAS set. After filtering the SNPs with low quality or with a read depth of less than three, 7,584 (19.6%) high quality SNPs remained. Among the 7,584 SNPs, 4,337 (57.2%) were transition mutations (C/T or G/A) and 3,247 (42.8%) were transversion mutations (C/G, A/T, C/A, or T/G). The average SNP density was one SNP per 10 kb of transcript sequences. Twenty-one pairs of primers were designed flanking 275 SNPs and 17 of them amplified specific bands (Table B3) and were successfully sequenced. In total, 28 (13.1%) SNPs contained in five different (29.4%) SNP amplicons were validated between the Mexico and Brazil accessions using sequencing (Table 5, Table B9). These validated SNPs had a read depth of 4 or more than ten when called computationally.

## 4. Discussion

### 4.1. Jatropha Transcript Sequencing and Assembly

In this study, we performed a de novo assembly of jatropha transcriptome in order to discover novel transcripts that might not have been discovered if we followed a map-based assembly on the current jatropha genome (version 4.5) [14] which covers 71% of the jatropha genome with 39,277 contigs (over 500 bp) and a N50 of 15,950 bp. The de novo assembled UAS set with an average length of 1,381 bp, which was longer than those in previous jatropha transcriptome studies, 916 bp, [17] and 789 bp [22], and transcriptome studies of other plants using the 454 sequencing platform (*Panax ginseng*, 559 bp [64]; blueberry, 933 bp [65]; Japanese larch, 667 bp [66]; switchgrass, 535 bp [67]; and sheepgrass, 607 bp [68]). In total, 18,391 (89.2%) contigs in our dataset were longer than 500 bp, and 12,008 (58.2%) contigs were longer than 1000 bp. The long assembled contigs may result from the long average read length, high sequencing depth, and a combination of assembler and cluster collapsing isoforms and removing redundant short sequences. A total of 21,418 UASs are full-length cDNAs, accounting for more than half of the genes predicted in the jatropha genome [14, 15] indicating fair quality of sequencing and assembling in our study.

Various de novo assembly algorithms have already been released, and each may model different portions of the true transcriptome with different accuracies [69, 70]. De novo assembly of our jatropha transcript sequences using Newbler v2.8 revealed that approximately 88.3% of the reads were assembled into contigs, which is comparable to the rates observed in other studies (American ginseng, 93.1% [71]; jatropha, 81.6% [17]; lentil, 91.9% [20]; and blueberry, 90.8% [65]). RT-PCR and Sanger sequencing of PCR products from 15 selected contigs verified the fidelity of the assembly. Ten longest contigs that were checked individually were full-length cDNAs with CDS and most of them specifically aligned to unique proteins with high sequence identity, suggesting a good assembly in our study. There are still some unassembled reads that remain as singletons in the UAS set (Table 1), which could have resulted from artifacts, sequencing error, low-abundant transcripts, simple repeat regions, or limitations of assembler algorithm [72, 73]. After checking the annotation of the singletons, we noticed that the majority of the unassembled reads or singletons have hits in the NCBI databases suggesting that they are most likely rare genes.

### 4.2. Enrichment of Current Jatropha Genomic Database

Hirakawa et al. [14] assembled previous jatropha ESTs together and predicted 30,203 genes out of which 25,433 were complete. By aligning to the jatropha CDS database, we found 23,264 new UASs in our database, which enriched the current EST database significantly. More than 21,418 full-length cDNAs identified in our study can be applied in analyzing functions of individual genes through gene transformation and other reverse genetic approaches.

A comprehensive annotation of the UAS set revealed diverse functional categories of the genes in the UAS set with most of them involved in cellular metabolism, which can be further characterized with specific biological experiments. Our annotations provide a great foundation and reference for designing those experiments. They also provide the basis for analyses of differential gene expression in jatropha cells submitted to microgravity. Genetic variability in jatropha is essential for breeding programs, selection of superior individuals, and consequently for assisting in the genetic improvement of jatropha [6]. For instance, the discovery of the transcription factors will facilitate further investigation of the diverse gene regulatory networks in jatropha. Several oil biosynthetic pathways were also identified, which are important in jatropha oil composition studies, although fewer genes were involved in these pathways than previously reported [15, 17], mostly due to the sampling of leaves. In addition, jatropha as a tropical plant is sensitive to cold stress and cold tolerance in jatropha might be a key for allowing the expansion of cultivation areas into more temperate climates. The mechanisms of how jatropha responds to cold stress need to be studied for the possibility of inducing or manipulating cold tolerance in jatropha. Previous studies have shown that plants undergo different changes in response to cold stress, which include physiological changes, activation of antioxidant salvage system, gene expression, and protein changes [74]. We found that jatropha does possess some genes common to cold stress responses (Table 3). Specifically, previous studies have shown that DREB1 expression could be upregulated by both cold stress and circadian clock [75]. HOS1, ICE1, and CBF1 were also found to be differentially expressed during cold stress in jatropha [22]. These orthologs are based on sequence similarity and their specific functions in jatropha need to be verified. These cold-responsive-gene orthologs could provide a reference for further cold stress response analysis in jatropha by overexpressing or knocking out the genes to understand the cold response network in jatropha.

For the KEGG pathway analysis, 1,637 UASs were mapped to 10 human disease categories including cancers, neurodegenerative diseases, cardiovascular diseases, and infectious diseases (Table B6). In order to check if this was due to contamination, we randomly picked one UAS from each category (10 UASs in total) and checked their functions. Most of these sequences were mapped to plant proteins with very basic functions, such as kinase, phosphatase, and transcription factors for basic levels of function in various pathways (Table B10). For example, contig 19405 mapped in human disease is an ortholog of K04371 (extracellular signal-regulated kinase 1/2), which was found in 77 pathways including MAPK signaling pathway, toll-like receptor signaling pathway, and pathways in cancer. Thus, these mapped human disease pathways suggest some nonspecificity of the category naming in KEGG instead of sample contamination.

### 4.3. SSR and SNPs from the Jatropha Transcriptome

We have discovered a large number of SSRs and SNPs for jatropha in this study that can be utilized by the breeding community for genetic improvement of jatropha. This complements previous studies on jatropha that were limited to 786 SSRs [76] and 432 EST-SSRs [77]. The dinucleotide SSR types discovered in this study had a higher frequency than other types of SSRs, which is consistent with previous studies [78]. The most abundant dinucleotide SSR type was AG/CT (45.8%), followed by AT/AT (17.0%) and AC/GT (5.2%) (Table B8). These numbers are slightly lower than previous studies in jatropha, but the trend is consistent [76, 77]. AG/CT was also found to be the most abundant dinucleotide type in other major crops, such as wheat, rice, maize, and soybean [79]. Among the trinucleotide SSR types, AAG/CTT (8.3%) was the most frequent, which was in agreement with other studies [76–78]. Dicots and monocots have different ratios of trinucleotide and dinucleotide. Dinucleotide SSRs are usually the most abundant types in dicots while trinucleotide SSRs are the most abundant type in monocots [76, 78, 79]. For the trinucleotide SSRs, the motif AAG/CTT is abundant in dicots [79]; however, the most abundant type of motif is different in different monocots. For example, AAC/GTT is the most common motif in wheat, AGG/CCT in rice and soybean, and CCG/CGG in barley and maize [79, 80]. The functions of these trinucleotide repeats are not fully understood, but some of them are found to be related to genes with certain functions. For example, CCG repeats were found to be involved in stress resistance, transcription regulation, metabolic enzyme biosynthesis, and signal transduction. AAC repeats were found to be related to the storage proteins glutenin and gliadin, important agronomical traits of wheat. This knowledge can be utilized to characterize novel trinucleotide repeat-containing genes when the relationship between particular trinucleotide repeats and specific functional categories of genes are established [79].

The total SSR amplification rate was 77.1%, which was comparable to a couple of previous studies in jatropha (82.82% [77]; 78% [76]), and slightly higher than another study (55.85%) [46]. The amplification rate in this study has demonstrated the success of SSR discovery and SSR primer design. Lack of amplification of the remaining primers might be due to (1) the primer sequence being in the exon-exon junction, (2) a large intron between exons to be amplified, and (3) sequencing error [81].

Though the main purpose of the amplification of the selected SSRs was to validate the SSR, we evaluated the SSR polymorphism using six selected germplasm accessions. The polymorphic rate of 19.8% in this study was much lower than previous studies in jatropha: 72.39% [77]; 44.63% [46]; 57.0% [76]. The low polymorphism rate might be because of the low genetic background of the jatropha accessions. Jatropha varieties from China, India, Africa, and Brazil had lower genetic variation than the varieties from Mexico [82]. Yue et al. [83] found no genetic variation among 278 individuals of jatropha collected from four continents at 29 loci using sequencing. In this study, all the polymorphic bands came from the Mexico accession, which is consistent with previous studies [82, 84]. Ambrosi et al. [84] analyzed 27 accessions of jatropha from different geographic locations in the world and found that the germplasms had limited genetic variation with the exception of accessions from Mexico.

Even though SNP discovery from EST sequences has been successfully applied in a large number of plants, such as soybean [85], alfa-alfa [86], black cottonwood [87], and cassava [88], limited studies have been reported in jatropha. In 2012, a total of 2,484 SNP markers were developed using 61 genotypes [47]. Among them, 47 out of 60 selected SNPs showed polymorphism. In our study, we sampled RNA from six jatropha genotypes, which showed significant phenotypic variation in the field and covered wide geographic areas, including three continents (Central America, Africa, and Asia) and one island in the South Pacific Ocean. Therefore, the sequence data set covered a large and diverse genetic pool and will be a potential resource for discovering transcript sequence variations. A total of 7,584 SNPs were called and the SNP validation rate was 13.1%. Since most of the validated SNPs had a read depth of more than ten, a read depth of 10 is recommended for jatropha SNP calling. The results of this study enriched the genetic tool box and current jatropha genomic database and provided SSR and SNP marker resources that can be utilized for genetic studies and molecular breeding in jatropha. These results are also essential for the evaluation of differential gene expression in jatropha cells submitted to microgravity, which may assist with breeding and genetic improvement programs for jatropha.

## Supplementary Material

Appendix A Fig A1 Classification distribution of UASs annotated by blasting eukaryotic orthologous groups (KOG) Fig A2 Pathway classification distribution of UASs annotated in KEGG database Fig A3 Fatty acid biosynthesis pathway. Appendix B Table B1 PCR confirmation of the assembled contigs from Newbler Table B2 List of SSR primers ordered for validation Table B3 List of SNP primers ordered for validation Table B4 Annotation and size of the top 10 longest contigs in the UAS set Table B5 Number of UASs aligned with protein and nucleotide sequences in Arabidopsis, castor bean and cassava Table B6 Number of UAS annotated involved in KEGG pathways Table B7 Thirty selected cold stress regulated genes Table B8 SSR motif types and numbers detected in the jatropha 454 databases Table B9 Details of validated SNPs Table B10 Ten selected jatropha UASs involved in human disease related pathways by KEGG pathway mapping.

## Figures and Tables

**Table 1:** Summary statistics of the transcript sequence reads assembly.

	Raw sequence	Assembled contigs (Newbler)	UASs (>100 bp) (Newbler + CD-HIT)	Contigs (>100 bp) (Newbler + CD-HIT)
Number of sequences	1,714,433	27,897	115,611	20,624
Min length	40	1	100	100
Max length	956	11,929	11,929	11,929
Median length	464	839	491	1168.5
Average length	441.2	1082.2	621.0	1380.9
N25	—	2,481	1,435	2,554
N50	—	1,679	556	1,746
N75	—	1,036	471	1,150
N90	—	607	396	751
GC%	41.67	40.45	39.54	40.56

**Table 2:** Identified UASs involved in oil biosynthesis and oil metabolic pathways.

Name	Definition	EC	KO
Fatty acid biosynthesis			
K01964	Acetyl-CoA/propionyl-CoA carboxylase	6.4.1.2; 6.4.1.3	K01964
accC	Acetyl-CoA carboxylase, biotin carboxylase subunit	6.4.1.2; 6.3.4.14	K01961
fabH	3-Oxoacyl-[acyl-carrier-protein] synthase III	2.3.1.180	K00648
fabG	3-Oxoacyl-[acyl-carrier protein] reductase	1.1.1.100	K00059
fabA	3-Hydroxyacyl-[acyl-carrier-protein] dehydratase	4.2.1.59	K01716
fabZ	3-Hydroxyacyl-[acyl-carrier-protein] dehydratase	4.2.1.59	K02372
fabI	Enoyl-[acyl-carrier protein] reductase I	1.3.1.9; 1.3.1.10	K00208
fabD	[Acyl-carrier-protein] S-malonyltransferase	2.3.1.39	K00645
DESA1	Acyl-[acyl-carrier-protein] desaturase	1.14.19.2	K03921
FATA	Fatty acyl-ACP thioesterase A	3.1.2.14; 3.1.2.-	K10782
FATB	Fatty acyl-ACP thioesterase B	3.1.2.14; 3.1.2.-	K10781
Linoleic acid metabolism			
PLA2G, SPLA2	Secretory phospholipase A2	3.1.1.4	K01047
LOX1_5	Linoleate 9S-lipoxygenase	1.13.11.58	K15718
LOX2S	Lipoxygenase	1.13.11.12	K00454

**Table 3:** Identified UASs involved in cold stress responses.

Gene	Protein	UAS hits
HOS1	E3 ubiquitin-protein ligase HOS1	Contig 15459
ICE1	Transcription factor ICE1	Contig 18116, contig 17481
ICE2	Inducer of CBF expression 2	Contig 18116, contig 17481
CBF1/DREB1b	Dehydration-responsive element-binding protein 1B	Contig 05683 contig 19154
RAV1	AP2/ERF and B3 domain-containing transcription factor RAV1	Contig 09872, contig 21221
Rd22	Dehydration-responsive protein RD22	Contig 20575

**Table 4:** Summary statistics of SSR detection and validation.

	Di-	Tri-	>3	Total
SSRs in the database	8,175	3,485	384	12,044
Ordered SSR primer pairs	88	174	—	262
Amplified SSRs	60	142	—	202 (77.1%)
Polymorphic SSRs	11	22	—	33
Polymorphic rate	18.3%	15.5%	—	16.3%

**Table 5:** Summary statistics of SNP detection and validation.

	Depth		Number
SNPs discovered in the database	0		38,693
	≥4		7,584
	≥10		4,767
Chosen SNPs for validation	4–10		96
	11–20		90
	21+		89
Primers designed flanking the chosen SNPs		21	
Total expected SNPs chosen		275	
Amplified primers for Sanger sequencing		19	
SNPs for Sanger sequencing		240	
Primers succeeded for sequencing		17	
SNPs succeeded for sequencing		214	
Primers validated containing SNPs		5 (29.4%)	
Matched SNPs with sequencing results		28 (13.1%)	
