# Supplementary material for: Enriching Genomic Resources and Marker Development from Transcript Sequences of Jatropha curcas for Microgravity Studies
Source: Int J Genomics. 2017 Jan 5;2017:8614160. doi: 10.1155/2017/8614160 (PMC5244023; doi:10.1155/2017/8614160)
Supplement: Supplementary file 1 — Appendix A Fig A1 Classification distribution of UASs annotated by blasting eukaryotic orthologous groups (KOG) Fig A2 Pathway classification distribution of UASs annotated in KEGG database Fig A3 Fatty acid biosynthesis pathway. Appendix B Table B1 PCR confirmation of the assembled contigs from Newbler Table B2 List of SSR primers ordered for validation Table B3 List of SNP primers ordered for validation Table B4 Annotation and size of the top 10 longest contigs in the UAS set Table B5 Number of UASs aligned with protein and nucleotide sequences in Arabidopsis, castor bean and cassava Table B6 Number of UAS annotated involved in KEGG pathways Table B7 Thirty selected cold stress regulated genes Table B8 SSR motif types and numbers detected in the jatropha 454 databases Table B9 Details of validated SNPs Table B10 Ten selected jatropha UASs involved in human disease related pathways by KEGG pathway mapping. [file 8614160.f1.zip › 8614160.f1/Fig A1_IJG_1815049.docx]

**Appendix A**

**Fig A1** Classification distribution of UASs annotated by blasting eukaryotic orthologous groups (KOG)

INFORMATION STORAGE AND PROCESSING

K: Transcription

J: Translation, ribosomal structure and biogenesis

A: RNA processing and modification

L: Replication, recombination and repair

B: Chromatin structure and dynamics

CELLULAR PROCESSES AND SIGNALING

O: Posttranslational modification, protein turnover, chaperones

T: Signal transduction mechanisms

U: Intracellular trafficking, secretion, and vesicular transport

D: Cell cycle control, cell division, chromosome partitioning

Z: Cytoskeleton

M: Cell wall/membrane/envelope biogenesis

V: Defense mechanisms

Y: Nuclear structure

W: Extracellular structures

N: Cell motility

METABOLISM

G: Carbohydrate transport and metabolism

E: Amino acid transport and metabolism

I: Lipid transport and metabolism

C: Energy production and conversion

Q: Secondary metabolites biosynthesis, transport and catabolism

P: Inorganic ion transport and metabolism

F: Nucleotide transport and metabolism

H: Coenzyme transport and metabolism

POORLY CHARACTERIZED

R: General function prediction only

S: Function unknown
