# Supplementary material for: Enriching Genomic Resources and Marker Development from Transcript Sequences of Jatropha curcas for Microgravity Studies
Source: Int J Genomics. 2017 Jan 5;2017:8614160. doi: 10.1155/2017/8614160 (PMC5244023; doi:10.1155/2017/8614160)
Supplement: Supplementary file 1 — Appendix A Fig A1 Classification distribution of UASs annotated by blasting eukaryotic orthologous groups (KOG) Fig A2 Pathway classification distribution of UASs annotated in KEGG database Fig A3 Fatty acid biosynthesis pathway. Appendix B Table B1 PCR confirmation of the assembled contigs from Newbler Table B2 List of SSR primers ordered for validation Table B3 List of SNP primers ordered for validation Table B4 Annotation and size of the top 10 longest contigs in the UAS set Table B5 Number of UASs aligned with protein and nucleotide sequences in Arabidopsis, castor bean and cassava Table B6 Number of UAS annotated involved in KEGG pathways Table B7 Thirty selected cold stress regulated genes Table B8 SSR motif types and numbers detected in the jatropha 454 databases Table B9 Details of validated SNPs Table B10 Ten selected jatropha UASs involved in human disease related pathways by KEGG pathway mapping. [file 8614160.f1.zip › 8614160.f1/Fig A2_IJG_1815051.docx]

**Fig A2** Pathway classification distribution of UASs annotated in KEGG database

KEGG Category
